# Characterization of HIV-1 CRF02_AG/A3/G unique recombinant forms identified among children in Larkana, Pakistan

**DOI:** 10.3389/fcimb.2023.1284815

**Published:** 2023-10-30

**Authors:** Abdur Rashid, Li Kang, Feng Yi, Fatima Mir, Yimam Getaneh, Yiming Shao, Syed Hani Abidi

**Affiliations:** ^1^ School of Medicine, Nankai University, Tianjin, China; ^2^ State Key Laboratory for Infectious Disease Prevention and Control, National Center for AIDS/STD Control and Prevention, Chinese Center for Disease Control and Prevention, Beijing, China; ^3^ College of Life Sciences, Nankai University, Tianjin, China; ^4^ Department of Pediatric and Child Health, Aga Khan University, Karachi, Pakistan; ^5^ Ethiopian Public Health Institute, Addis Ababa, Ethiopia; ^6^ Changping Laboratory, Beijing, China; ^7^ Department of Biological and Biomedical Sciences, Aga Khan University, Karachi, Pakistan; ^8^ Department of Biomedical Sciences, School of Medicine, Nazarbayev University, Astana, Kazakhstan

**Keywords:** HIV-1, genetic recombination, phylogeny, children, Larkana, Pakistan

## Abstract

Co-circulation of different human immunodeficiency virus type 1 HIV-1 subtypes among infected populations can lead to the generation of new recombinants. In Pakistan, subtype A1 and CRF02_AG are the dominant strains circulating among key populations. The high prevalence of new HIV infections among the key populations highlights the possibility of recombination between the dominant strains, which can lead to the generation of new recombinants. Here, we identified a recombinant cluster composed of CRF02_AG, sub-subtype A3, and subtype G among HIV-infected children in Larkana. For the study, 10 retrospectively collected samples, with recombination signals in the *pol* gene, were used to perform a near full-length genome NFLG sequencing. Of the 10 samples, NFLG was successfully sequenced from seven samples. Phylogenetic analysis of the seven NFLGs showed that all recombinants formed a distinct monophyletic cluster and were distinct from known HIV-1 circulating recombinant forms CRFs. Recombination analyses showed that all seven NFLGs shared a similar recombinant structure consisting of CRF02_AG, sub-subtype A3, and subtype G, with a sub-subtype A3 fragment inserted into *pol* and *vif* regions spanning from (HXB2: 4218-5518), and a subtype G fragment inserted into *vpu*, *rev*, *tat* and *env* regions spanning from (HXB2: 5957-8250) of the CRF02_AG backbone. The identification of unique recombinant forms may indicate the presence and transmission of several co-circulating lineages in Larkana, giving rise to newer CRFs. This study also highlights the importance of continuous molecular surveillance to fully understand HIV-1 genetic diversity in Pakistan, particularly in Larkana, which is the epicenter of HIV outbreaks.

## Introduction

Human Immunodeficiency Virus Type 1 (HIV-1) is divided into four distinct phylogenetic groups: M, N, O, and P (Gao et al., 1999). HIV-1 M-group lineages, which dominate the HIV-1 pandemic and are responsible for 95% of global HIV infections, are further divided into nine subtypes: A-D, F-H, J, and K (Robertson et al., 2000; Rashid et al., 2022). High mutation rate and genomic recombination are hallmarks of HIV-1 infection, which leads to extraordinary genetic variability and evolution (Smyth et al., 2012; Olabode et al., 2022). Recombination is a major facilitator through which HIV-1 increases its genetic diversity, which is driven by template switching during its replication cycle (Cromer et al., 2016) and by co-infection with multiple strains at the cellular (Neher and Leitner, 2010) and host levels (Redd et al., 2013).

In the late 1980s, the first evidence of an HIV-1 recombinant genome was reported (Li et al., 1988). Recombination between HIV-1 strains of the same or different clades may result in the generation of important founder strains (Zhang et al., 2010). HIV-1 recombinants are classified into circulating recombinant forms (CRFs) and unique recombinant forms (URFs). CRF is a mosaic of two or more HIV-1 subtypes with the same pattern of recombination breakpoints that have been identified in at least three epidemiologically unlinked individuals, whereas URF refers to recombinant that do not meet the CRF criterion but only identified in one individual [4]. Currently, more than 121 CRFs and uncountable URFs have been submitted to the Los Alamos HIV Database (http://www.hiv.lanl.gov). Globally, 22.8% of HIV-1 strains sequenced are inter-subtype recombinants, of which 73.3% are CRFs and 26.7% are URFs (Hemelaar et al., 2020).

The first cases of HIV infection in Pakistan were reported in 1987 among repatriated Pakistanis from the Gulf States (Khanani et al., 1988). Pakistan is among the countries where the estimated number of new HIV infections has increased every year. In the last decade, Pakistan has seen a 78.5% increase in new HIV infections (https://www.aidsdatahub.org). In Pakistan, HIV-1 genotypes exhibit considerable heterogeneity, with HIV-1 subtypes A1 and CRF02_AG being the predominant strains, followed by B, C, D, G, CRF35_A1D, CRF01_AE, and CRF56_cpx (http://www.hiv.lanl.gov). The co-circulation of distinct strains among HIV-1 key population groups can increase the chances of co-infection and may have led to the generation of new recombinants, such as DG (Tariq et al., 2020), A1G, CRF02A1, and A1D (Chen et al., 2016; Cholette et al., 2020).

Larkana is a rural district located in Sindh Province, with a driving distance of 452 km from Karachi, Pakistan. Since early 2000, Larkana has experienced three HIV outbreaks in 2003, 2016, and 2019 (Zaid and Afzal, 2018; Zaid et al., 2019). The 2019 outbreak predominantly involved children, where more than 1,000 children were found to be seropositive for HIV-1 [20]. A molecular epidemiological study of the Larkana outbreak, performed using the pol sequences sampled from 344 infected children, showed CRF02_AG and subtype A1 as the dominant strains in Larkana, while a transmission cluster of 10 unknown recombinants was identified that were not reported previously (Abidi et al., 2021; Abidi et al., 2022). To fully understand the recombination patterns in the 10 unknown recombinant strains, we performed near full-length genome (NFLG) sequencing, followed by phylogenetic analysis of the amplified sequences.

## Materials and methods

### Study population

This study was performed on 10 samples retrospectively collected during the 2019 outbreak investigation (April 2019). These samples were selected based on the presence of a recombination signal in the pol region in the preliminary phylogenetic analysis (Abidi et al., 2021; Abidi et al., 2022). All subjects were registered for HIV care at the Pediatric Treatment Center at Shaikh Zayed Children’s Hospital. This center was established by Sindh AIDS Control Program in response to the 2019 HIV-1 outbreak. A unique laboratory identification number (AKULO_295, AKULO_301, AKULO_187, AKULO_81, AKULO_194, AKULO_173, AKULO_1, AKULO_353, AKULO_329, and AKULO_248) was given to each sample to ensure the confidentiality of the study participants. Written informed consent was obtained from the parents/guardians, and if the child was able to understand the study procedures, a written assent was obtained (Abidi et al., 2022). This study was approved by the Institutional Ethics Committee of the School of Medicine, Nankai University, Chinese Disease Control Center, China CDC, and Aga Khan University (AKU ERC# 2019-1536-4200). All experiments were performed in accordance with approved guidelines and regulations.

### Near full-length genome amplification and sequencing

All 10 samples were used for near full-length genome sequencing, following previously reported methods (Rousseau et al., 2006; Li et al., 2012). The first-round PCR was performed in 25μl of the final reaction mixture with 3μl DNA template and 2ul of the primer mix (**Supplementary Table S1**). The second-round PCR was performed in 50μl of the final reaction mixture with a 2μl aliquot of the first round as a template and 4ul of the primer mix (**Supplementary Table S1**). The thermocycle conditions for both rounds of PCR were the same: initial PCR activation at 94°C for 3min, followed by 35 cycles of denaturation at 94°C for 20sec, annealing at 60°C for 30s, extension at 68°C for 4min, and final extension at 68°C for 10min. The final PCR amplicons were visualized on a 1% agarose gel with a ladder size of 15,000 bp to confirm the nested PCR product. Amplified amplicons were sequenced on an ABI 3730XL sequencer using BigDye terminators (Applied Biosystems, Foster City, CA, USA).

### HIV-1 near full-length genome sequence analysis

Of the 10 samples, seven NFLGs were successfully sequenced from 7 samples. NFLG chromatogram data were spliced and assembled using Sequencher v5.4.6 (Gene Codes Corporation, Ann Arbor, MI, USA) and manually edited. The final NFLG sequences were aligned with the 2020 HIV-1 M group subtypes and CRFs reference sequences downloaded from the Los Alamos HIV Database (http://www.hiv.lanl.gov), using MAFFT v7 (Katoh et al., 2019), and manually edited using AliView v1.17.1 (Larsson, 2014) and BioEdit v7.2.5 (Hall, 1999) software. The aligned sequences were used to generate a maximum likelihood phylogenetic tree using IQ-Tree v2.0 (Nguyen et al., 2015), with a general time-reversible plus gamma (GTR+G) model of nucleotide substitution and the Shimodaira-Hasegawa approximate likelihood ratio test (SH-aLRT) measure of branch support and 1,000 bootstrap replicates for phylogenetic cluster robustness. ML phylogeny was visualized and edited in Figtree v1.4.4 (http://www.tree.bio.ed.ac.uk/software/figtree/).

### HIV-1 recombination analysis

The seven NFLG sequences of AKULO recombinants were analyzed to detect recombination using the two recombination tools, RIP (Recombination Identification Program) and jumping profile hidden Markov model (jpHMM) available at the Los Alamos HIV Database (http://www.hiv.lanl.gov). To define the recombination breakpoints in the AKULO sequences, a recombinant bootscan was implemented in Simplot version 3.5.1, using the HIV-1 M group subtype reference sequences, with the following parameters: window size of 500 bp and a step size of 20 bp, and the neighbor-joining method using the Kimura 2-parameter model with 100 replicates. Subsequently, similarity plot analysis for NFLGs was conducted in Simplot v3.5.1, using the aforementioned parameters and HIV-1 subtypes consensus reference alignment downloaded from the Los Alamos HIV Database (http://www.hiv.lanl.gov).

Furthermore, for subregion confirmation, phylogenetic trees were constructed using IQTree to confirm the inter-subtype recombination breakpoints and HIV-1 subtype within each segment of the AKULO-recombinants using HIV-1 group M subtype reference sequences. For each segment of the recombinant, a ML phylogenetic tree was constructed with a general time-reversible plus gamma (GTR+G) model of nucleotide substitution and the Shimodaira-Hasegawa approximate likelihood ratio test (SH-aLRT) for testing of branch support with 1000 replicates, and a bootstrap value of ≥80% was considered definitive. All sub-genomic phylogenies were visualized in Figtree v1.4.4 (http://www.tree.bio.ed.ac.uk/software/figtree/). Finally, the genomic structure of the AKULO-recombinant was generated using the Recombinant HIV-1 Drawing Tool available online in the HIV LANL Database (http://www.hiv.lanl.gov).

### Drug resistance mutations and co-receptor tropism analyses

Antiretroviral drug resistance mutations were determined using Stanford University’s HIV drug resistance database algorithm v9.4 (https://hivdb.stanford.edu), while HIV-1 co-receptor usage was determined using the gp120 V3 loop amino acid sequence of individual study participants using the Geno2pheno v3.4 tool (https://www.geno2pheno.org).

## Results

### Demographic information of the study population

We used 10 retrospectively collected samples from HIV-1-positive children from Larkana, Pakistan, with a signal of recombination. The demographic information of the study participants subjected to the NFLG analysis is shown in (**Table 1**). All study participants’ ages at the time of sampling were less than 10 years, with a median age of 3 years (range 1.4-9 years). Among the 10 study participants, 6 were male and 4 were female. At the time of sampling, two participants hadn’t started antiretroviral therapy, and the remainder had just recently initiated antiretroviral therapy, comprising zidovudine, lamivudine, and nevirapine [18]. Of the 10 study participants, (n = 8) reported being infected in the Larkana district, the epicenter of the 2019 outbreak, while (n = 2) participants reported being infected in the Shikarpur district, located 72 km away from Larkana district. All cases were identified as hospital-acquired infections (HAIs)(Abidi et al., 2021; Abidi et al., 2022).

**Table 1:** Demographic information of the 10 children living with HIV.

Sample ID	Gender	Sampling Time	Age (Years)	ART Regimen	Therapy Duration	Location
AKULO_301	Female	05-08-2019	7.3	LNZ	2 months	Sindh 77150 (Larkana)
AKULO_81	Female	30-05-2019	0.8	LNZ	20 days	Ratodero (Larkana)
AKULO_194	Female	27-06-2019	1.4	LNZ	1 month	Ratodero (Larkana)
AKULO_329	Female	19-08-2019	3	Naïve	N/A	Sindh 77150 (Larkana)
AKULO_173	Male	24-06-2019	1.9	LNZ	1 month	Ratodero (Larkana)
AKULO_1	Male	17-05-2019	1.6	LNZ	11 days	Ratodero (Larkana)
AKULO_248	Male	10-07-2019	9	Naïve	N/A	Sindh 77150 (Larkana)
AKULO_295	Male	02-08-2019	3.2	LNZ	1.75 months	Abid Markhiani (Shikarpur)
AKULO_187	Male	26-06-2019	4	LNZ	1.25 months	Ratodero (Larkana)
AKULO_353	Male	21-08-2019	3	LNZ	2 months	Khan Jalbani (Shikarpur)

LNZ, lamivudine, nevirapine and zidovudine; N/A, information not available.

### Phylogenetic analysis

Of the 10 AKULO-recombinant samples, we successfully sequenced NFLGs from seven samples. The NFLG sequences from the seven study participants were ≥8000 bp long (**Table 2**), ranging from the 5`-gag region covering *pol, vif, vpr, vpu, rev, tat, env, nef* to the 3`-LTR (nucleotides 643-9615; in reference to HXB2 nucleotide position).

**Table 2:** Seven NFLG sequences of AKULO recombinants.

Sample ID	Length (nucleotide)
AKULO_248	8947
AKULO_301	8928
AKULO_173	8934
AKULO_329	8939
AKULO_81	8951
AKULO_1	8934
AKULO_173	8934

AKULO, Aga Khan University Larkana Outbreak.

**Figure 1:**
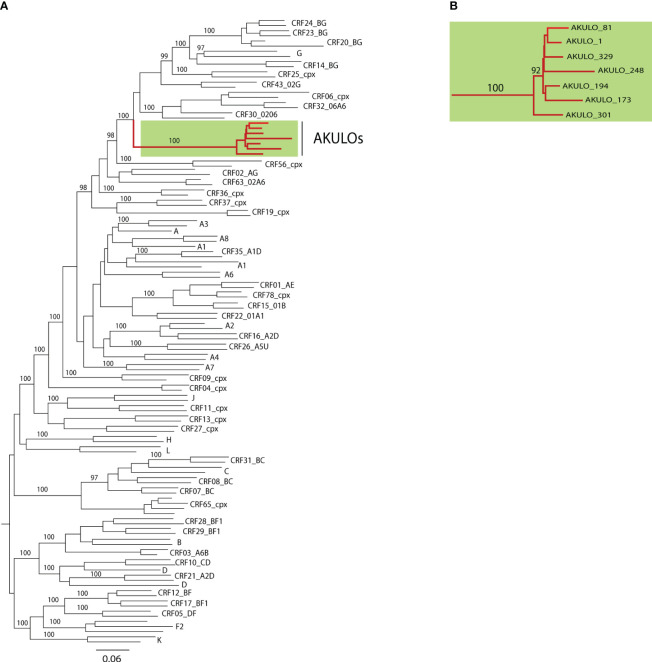
Phylogenetic analysis based on the 7 near full-length genomic sequences (HXB2 position 643-9615). **(A)** Maximum likelihood phylogenetic tree consisting of 7 AKULO-recombinants NFLGs and all NFLGs reference dataset (2020) of HIV-1 group M subtypes and CRFs downloaded from LANL database (https://www.hiv.lanl.gov). Nodes significance in the tree was assessed by bootstrap analysis with 1000 replicates, and only those nodes with bootstrap values above ≥90% are shown **(B)** Cluster of NFLG sequences of the 7 AKULO-recombinants.

ML phylogenetic analysis of the seven AKULO NFLGs revealed that none of the seven AKULO NFLGs clustered with any HIV-1 reference subtype or known CRFs but clustered together and formed a distinct monophyletic branch with a bootstrap value of 100% (**Figures 1A, B**). Phylogenetic analysis indicated that the seven AKULO NFLGs are potentially new recombinant strains circulating in Larkana, Pakistan.

### Recombination analysis of AKULO-recombinants

**Figure 2:**
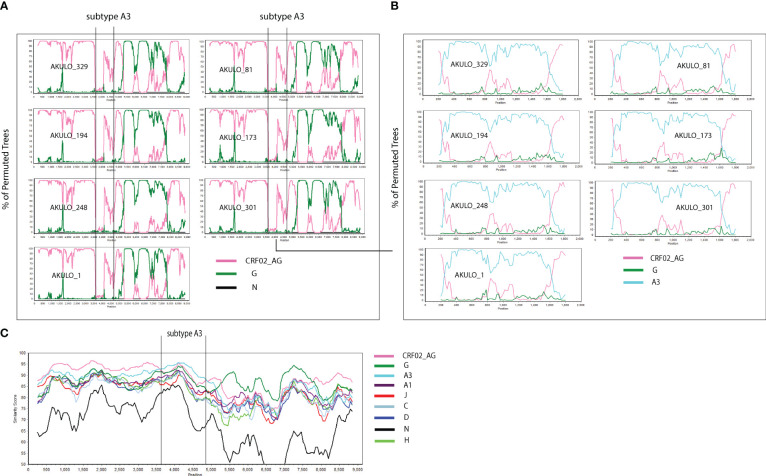
Simplot analyses of the 7 AKULO NFLGs recombinants sequences. **(A)** Bootscan analysis of the 7 AKULO NFLGs recombinants. **(B)** Bootscan analysis of the sub-subtype A3 fragment inserted into CRF02_AG backbone. **(C)** similarity plot analysis of AKULO NFLGs recombinants. In all bootscan and similarity plot analyses a window size of 500 bases and a step size of 20 bases along with HIV-1 subtypes reference alignment were used. The x axis shows the nucleotide positions, and the y axis on the bootscan analysis shows the % bootstrap values of the permuted trees, while the y axis on the similarity plot shows the % similarity against the HIV-1 subtypes reference sequences.

The near full-length genome analysis of the seven AKULO recombinants confirmed a unique recombination composed of CRF02_AG, subtype G, and sub-subtype A3 (**Figures 2A, B**). The recombination breakpoints were identified using RIP, jpHMM, bootscan, and similarity plot analysis. Although the sub-subtype A3 fragment was only identified with bootscan and similarity plot analyses. However, BLAST search and ML phylogenetic tree analyses confirmed strong sequence similarity (93%) to sub-subtype A3. To our knowledge, this is the second recombinant containing a fragment of sub-subtype A3, originally identified in Senegal (http://www.hiv.lanl.gov).

**Figure 3:**
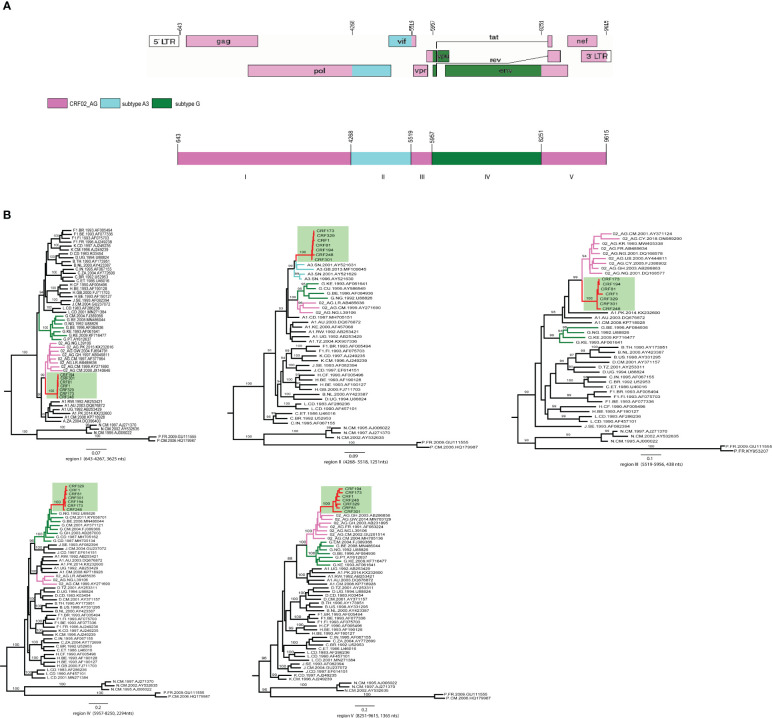
The analysis of recombination breakpoints in the 7 AKULO NFLGs sequences. **(A)** Near full-length genomic structure of the AKULO-recombinants (HXB2: 643-9615) was generated using the Recombinant HIV-1 Drawing Tool online available at (Recombinant Genome Drawing Tool (lanl.gov), The mosaic fragments in the AKULO NFLGs recombinants are colored as follows: CRF02_AG =pink, sub-subtype A3 =light blue and subtype G =green. **(B)** ML phylogenetic trees constructed for each of the five mosaic fragments identified by bootscan and similarity plot analyses. The AKULO NFLGs recombinants are colored red, and the background is highlighted light green, while CRF02_AG, sub-subtype A3 and subtype G reference sequences in each tree are colored pink, light blue and green, respectively. The nucleotide position in each fragment has been numbered according to HIV-1 reference sequence HXB2 (K03455) nucleotides position.

The bootscan analysis showed that the seven AKLUO recombinants displayed the same recombination pattern and shared four recombination breakpoints that were not reported previously (**Figures 2A, B**). Similarity plot analysis revealed that the recombinant structure of AKULO NFLGs belonged to CRF02_AG, sub-subtype A3, and subtype G, with five fragments separated by four unique inter-subtype recombination breakpoints that were identical in all seven AKULO NFLGs (**Figure 2C**). The four inter-subtype recombination breakpoints between CRF02_AG, sub-subtype A3, and subtype G were located at nucleotide positions 4268nt in *pol*, 5519nt in *vif*, 5957nt in *vpu* and 8251nt in *env* (gp41) regions, with reference to the HXB2 nucleotide position (**Figure 3A**).

The genomic structure of AKULO recombinants depicted five mosaic fragments, which consist of three CRF02_AG, one sub-subtype A3, and one subtype G fragment (**Figure 3A**). The near full-length genome of the AKULO recombinant structure was 60.5% covered by CRF02_AG, 13.9% by sub-subtype A3, and 25.5% by subtype G. The genomic structure of the AKULO recombinants recombination pattern also showed that AKULO recombinants could be a second-generation recombinant form derived from recombination between CRF02_AG, sub-subtype A3, and subtype G.

The AKULO recombinant mosaic structure was determined using bootscan, and similarity plot analyses were further confirmed by maximum likelihood phylogenetic analysis of each mosaic fragment. The ML phylogenetic trees constructed for the five mosaic fragments confirmed the recombination breakpoints of the AKULO recombinants as follows: region I (HXB2:643-4267, 3625nts) CRF02_AG, region II (HXB2:4268-5518, 1251nts) sub-subtype A3, region III (HXB2:5519-5956, 438nts) CRF02_AG, region IV (HXB2:5957-8250, 2294nts) subtype G, and region V (HXB2:8251-9615, 1365nts) CRF02_AG (**Figure 3B**).

The sub-region phylogenetic analyses also showed that the AKULO recombinant backbone fragments of CRF02_AG I, III, and V clustering with CRF02_AG lineages from Cameroon, Ghana, Nigeria, France, and Pakistan, whereas the inserted fragments (sub-subtype A3 and subtype G) clustering with sub-subtype A3 lineage from Senegal and subtype G lineages from Nigeria, Cameroon, Ghana, and the Democratic Republic of the Congo. The recombinant structure of AKULO NFLGs was distinct from any known HIV-1 CRFs, and all seven NFLGs were obtained from HIV-1 infected children in Larkana, Pakistan, suggesting these to be a new HIV-1 recombinant form.

### Drug resistance and co-receptor tropism

Among the seven study participants, only two were drug-naïve, while five were on ART at the time of sampling. Of the seven *pol* sequences, only one sequence (AKULO_194) contained mutations, K103N, and V179L associated with resistance to NNRTIs.

HIV-1 coreceptor tropism analysis showed that all seven sequences were CCR5 usage variants. Of the seven sequences, five had a V3 loop crown motif consisting of GPGQ, whereas two had a V3 loop crown motif consisting of RPGQ and APGQ.

## Discussion

In this study, the characterization of seven NFLGs of CRF02_AG/A3/G recombinants sequenced from HIV-positive children revealed a mosaic structure of the recombinants that was distinct from known HIV-CRFs, with a pattern of recombination breakpoints that were consistent in all seven NFLGs sequences. These sequences also formed a distinct monophyletic cluster with a 100% bootstrap value designated to be a new unique recombinant form in Larkana, Pakistan (Robertson et al., 2000). In Pakistan, the distribution of HIV-1 genotypes is heterogeneous, with subtype A1 being the most prevalent strain, with a prevalence of 68.0%, followed by CRF02_AG with a prevalence of 16.5%.(http://www.hiv.lanl.gov). Both strains were introduced into Pakistan in 1989 and 1996, respectively [14]. However, CRF02_AG has exhibited a noticeable rise in prevalence across serval urban areas (Yaqub et al., 2019; Abidi et al., 2021). The CRF02_AG strain was first detected in 2009 among Afghan refugees in Karachi, Pakistan (Ansari et al., 2011), whereas subtype G was first detected in 2007 among MSM in Karachi, Pakistan (Khanani et al., 2011). Bayesian analysis of CRF02_AG suggested that the time to the most common recent ancestor (tMRCA) of CRF02_AG in Larkana Pakistan was approximately 2016 (95% HPD interval:2015-2017) (Abidi et al., 2021). Similarly, no information is available regarding the introduction of subtype G in Pakistan, although subtype G prevalence is gradually increasing in Pakistan (http://www.hiv.lanl.gov).

Recombination in HIV requires infection with distinct strains at a cellular level in a single host. Individuals infected with different strains of HIV have been reported, which implies that HIV infected individuals must have had multiple infections (Templeton et al., 2009). HIV-1 transmission in 2019 HIV outbreak was strongly linked to visits to health care facilities in Larkana. The root cause of these transmissions was primarily attributed to poor infection control practices, such as the reuse of contaminated syringes and blood transfusions, resulting in co-infection leading to recombination of different strains (Siddiqui et al., 2020; Abidi et al., 2021; Mir et al., 2021). CRF02_AG was the dominant strain detected in the 2019 HIV outbreak, along with other subtypes (subtype A1, G, D) (Abidi et al., 2021).Recombination analysis revealed that the unique recombinant structure of AKULO NFLGs comprised 60.5% CRF02_AG, 13.9% sub-subtype A3, and 25.5% subtype G (**Figure 3A**). Notably, during the 2019 Larkana HIV-1 outbreak, there was a predominance of CRF02_AG and several strains of subtype G (Abidi et al., 2021). These unique recombinant forms emerged through the recombination of pre-existing CRF02_AG and subtype G, whereas sub-subtype A3 has not been previously reported in Pakistan. Sub-subtype A3 was previously identified in Central and West Africa, where sub-subtype A3/CRF02_AG recombinants have also been reported (Meloni et al., 2004). Previous studies have reported the emergence of new recombinants 02A1 (Chen et al., 2016; Yaqub et al., 2019), and DG (Tariq et al., 2020) via co-infection and recombination, thereby influencing the nature of the HIV-1 epidemic in Pakistan.

HIV-1 drug resistance mutation analysis showed the presence of mutations K103N and V179L associated with resistance against NNRTIs, such as nevirapine, which is the first-line regimen used in Pakistan. Mutations in these codon positions have been previously reported in Pakistan (Shah et al., 2011; Abidi et al., 2021). K103N is the most commonly transmitted drug resistance mutation, and it reduces susceptibility to efavirenz and nevirapine by approximately 20 and 50-fold, respectively (https://hivdb.stanford.edu). The detection of such type of mutation that shows resistance to first-line regimen available in Pakistan is alarming, as it constrains treatment options.

HIV-1 enters host cells by interacting with CD4 and chemokine receptors (CCR5 or CXCR4). The HIV-1 gp120 V3 loop is a major determinant of co-receptor tropism. Analysis of the V3 loop of 7 CRF02_AG/A3/G recombinant strains showed these strains to be CCR5 usage variants, suggesting that these strains may be susceptible to the CCR5 antagonist Maraviroc (Palladino et al., 2015).

The strengths of the study include the identification of 7 unique recombinant forms through near full-length genome from samples collected during the 2019 HIV outbreak in Larkana, Pakistan. The study performed genetic characterization of the unique recombinant form using phylogenetic and recombination analysis. However, there are certain limitations of the study such as the relatively small sample of only 10 HIV-1 positive children, and its findings are limited to the Larkana region. Nonetheless, this study significantly contributes to our understanding of HIV-1 epidemiology and evolution worldwide. Additionally, due to the non-availability of information, the study doesn’t provide clinical characteristics for the children infected with these unique recombinant forms.

In conclusion, this study reports a unique recombinant form derived from CRF02_AG, sub-subtype A3, and subtype G. The recombinant structure consisted of a CRF02_AG backbone and inserted fragments of sub-subtype A3 and G subtype identified among HIV-infected children in Larkana, Pakistan. The identification of a new recombinant form may indicate the presence and transmission of several co-circulating lineages in Larkana, possibly contributing to the emergence of new circulating recombinant forms. This study also highlights the importance of continued molecular surveillance, especially when employing the NFLG approach, to fully understand the HIV-1 genetic diversity in Pakistan.

## Data availability statement

The datasets presented in this study can be found in online repositories. The names of the repository/repositories and accession number(s) can be found below: https://www.ncbi.nlm.nih.gov/genbank/, OR345456, OR345457, OR345458, OR345459, OR345460, OR345461, OR345462.

## Ethics statement

The studies involving humans were approved by Institutional Ethics Committee of the School of Medicine, Nankai University, Chinese Disease Control Center, China CDC, and Aga Khan University. The studies were conducted in accordance with the local legislation and institutional requirements. Written informed consent for participation in this study was provided by the participants’ legal guardians/next of kin. 

## Author contributions

AR: Formal Analysis, Methodology, Software, Writing – original draft. LK: Formal Analysis, Methodology, Software, Writing – original draft. FY: Data curation, Investigation, Methodology, Supervision, Writing – review & editing. FM: Data curation, Resources, Writing – review & editing. YG: Data curation, Methodology, Writing – review & editing. YS: Conceptualization, Data curation, Funding acquisition, Investigation, Methodology, Project administration, Resources, Supervision, Validation, Writing – review & editing. SA: Conceptualization, Investigation, Project administration, Resources, Supervision, Validation, Writing – review & editing.
