# Supplementary material for: Characterization of HIV-1 CRF02_AG/A3/G unique recombinant forms identified among children in Larkana, Pakistan
Source: Front Cell Infect Microbiol. 2023 Oct 30;13:1284815. doi: 10.3389/fcimb.2023.1284815 (PMC10642767; doi:10.3389/fcimb.2023.1284815)
Supplement: Supplementary file 1 [file Table_1.docx]

**Table S1. Primers used in this study**

| **Primer name** |  | **Sequence** | **Direction** | **Position** |
| --- | --- | --- | --- | --- |
| F1.5 | First-round | CCTTGAGTGCTTCAAGTAGTGTGTGCCCGTCTGT | Forward | 0538-0571 |
| F1.3 |  | CCTARTGGGATGTGTACTTCTGAACTT | Reverse | 5219-5193 |
| F2.5 |  | AGTGGCGCCCGAACAGG | Forward | 0634-0650 |
| F2.3 |  | ATCATCACCTGCCATCTGTTTTCCAT | Reverse | 5066-5041 |
|  |  |  |  |  |
| L1.5 | Second-round | 5-AAAAATTCAAAATTTTCGGGTTTATTACAG-3 | Forward | 4883-4912 |
| L1.3 |  | TGAAGCACTCAAGGCAAGCTTTATTGAGGC | Reverse | 9607-9636 |
| LOW |  | ACTACTTGAAGCACTCAAGGCAAGCTTTATTG | Reverse | 9611-9642 |
| L2.5 |  | 5-GGGTTTATTACAGRGACAGCAGAG-3 | Forward | 4900-4923 |
| L2.3 |  | TGAGGCTTAAGCAGTGGGTTCC | Reverse | 9591-9612 |

**First and second round PCR. Reference of the primers:** (Li, Z., He, X., Wang, Z., Xing, H., Li, F., Yang, Y., Wang, Q., Takebe, Y., & Shao, Y. (2012). Tracing the origin and history of HIV-1 subtype B' epidemic by near full-length genome analyses. *AIDS (London, England)*, *26*(7), 877–884. <https://doi.org/10.1097/QAD.0b013e328351430d>), (Rousseau, C. M., Birditt, B. A., McKay, A. R., Stoddard, J. N., Lee, T. C., McLaughlin, S., Moore, S. W., Shindo, N., Learn, G. H., Korber, B. T., Brander, C., Goulder, P. J., Kiepiela, P., Walker, B. D., & Mullins, J. I. (2006). Large-scale amplification, cloning and sequencing of near full-length HIV-1 subtype C genomes. *Journal of virological methods*, *136*(1-2), 118–125. <https://doi.org/10.1016/j.jviromet.2006.04.009>)
